# The subcellular localization-dependent dual role of NR4A1 in renal fibrosis: mechanisms and therapeutic perspectives

**DOI:** 10.3389/fphar.2026.1883285

**Published:** 2026-08-20

**Authors:** Xu Li, Yufeng Qiao

**Affiliations:** 1 The Nephrology Department of Shanxi Provincial People’s Hospital, Shanxi Medical University, Taiyuan, China; 2 The Nephrology Department of Shanxi Provincial People’s Hospital, Taiyuan, China

**Keywords:** dual role, Nr4a1, renal fibrosis, subcellular localization, targeted therapy

## Abstract

Renal fibrosis is a common pathological process underlying the progression of various kidney diseases to end-stage renal disease. It is characterized by the activation of renal interstitial fibroblasts and excessive deposition of the extracellular matrix (ECM). Continued progression can lead to irreversible loss of renal function, but effective targeted therapies are currently lacking. In recent years, nuclear receptor 4A1 (NR4A1) has been shown to bidirectionally regulate renal fibrosis. Nuclear NR4A1 exerts anti-fibrotic effects by inhibiting pathways such as TGF-β/Smad and NF-κB. However, upon pathological stimulation, NR4A1 translocates to the cytoplasm and mitochondria, where it promotes fibrosis by activating the NLRP3 inflammasome and p38 MAPK signaling and inducing mitochondrial damage. Thus, the functional outcome of NR4A1 is highly dependent on its subcellular localization. This review focuses on the dual anti-fibrotic and pro-fibrotic mechanisms of NR4A1 in renal fibrosis. Based on the regulation of its subcellular localization shifts, we explore targeted intervention strategies aimed at precisely stabilizing its nuclear protective function while blocking pathological nuclear export, thereby providing new directions for treating renal fibrosis.

## 1 Introduction

Chronic kidney disease (CKD) is characterized by progressive structural damage and functional impairment of the kidneys. Globally, approximately 788 million adults were living with CKD in 2023, corresponding to a prevalence rate of 14.2%, representing a substantial increase from the 378 million cases reported in 1990. In the same year, approximately 152 million CKD patients resided in China, representing the largest national CKD population worldwide (Mark et al., 2025). Renal fibrosis, a common pathological feature of progressive CKD, is characterized by excessive accumulation and deposition of extracellular matrix (ECM) components, tubulointerstitial injury, and glomerulosclerosis, ultimately leading to renal failure (Wang et al., 2026).

The orphan nuclear receptor 4A (NR4A) subfamily, a key member of the nuclear receptor superfamily, plays a critical role in maintaining cellular homeostasis and regulating diverse physiological processes. Among NR4A family members, NR4A1 is widely expressed across multiple tissues, including the heart, kidney, liver, lung, adrenal gland, thyroid, thymus, pituitary, testes, ovaries, muscle, and prostate. Through transcriptional regulation, it participates in a variety of biological processes such as cell proliferation, differentiation, apoptosis, autophagy, inflammation, and metabolism (Gao et al., 2024). In recent years, a dual regulatory role for NR4A1 has been demonstrated across various diseases. In cancer, NR4A1 can function as either a tumor suppressor or a tumor promoter, depending on the type and stage of the malignancy (Wang et al., 2025; Jin et al., 2025). NR4A1 can also influence the onset and progression of kidney diseases. Some studies suggest that NR4A1 may exert dual regulatory roles in renal fibrosis—both promoting and inhibiting fibrosis—although the underlying mechanisms remain to be fully elucidated (Wang et al., 2023). Therefore, this review summarizes recent advances in understanding the dual regulatory roles of NR4A1 in renal fibrosis. To this end, we performed a structured literature search of PubMed, Embase, and Web of Science using keywords including “NR4A1”, “Nur77”, “renal fibrosis”, and “subcellular localization”, covering publications up to July 2026. The underlying mechanisms are discussed with a focus on subcellular localization-dependent signaling pathways, aiming to provide new directions for targeting NR4A1 in the treatment of renal fibrosis.

## 2 Biological features of NR4A1

### 2.1 Structural features of NR4A1

The orphan nuclear receptor 4A (NR4A) subfamily, which belongs to the nuclear receptor (NR) superfamily, comprises nerve growth factor-induced gene B (NR4A1, Nur77, NGFI-B, TR3, or NAK), Nur-related protein 1 (NR4A2, Nurr1), and neuron-derived orphan receptor 1 (NR4A3, Nor1, or MINOR) (Weikum et al., 2018). NR4A1 is a 598-amino-acid protein with a molecular weight of approximately 64 kDa, and its structural features underlie its multifaceted regulatory roles in inflammation, metabolism, cell proliferation, and apoptosis. NR4A1 harbors the canonical nuclear receptor domain architecture, namely an N-terminal transcription-activating domain (TAD), a central DNA-binding domain (DBD), and a C-terminal ligand-binding domain (LBD) (Kiriyama et al., 2025). The TAD, located at the N-terminus, initiates downstream gene transcription by interacting with basal transcription factors, coactivators, or other transcriptional regulators. The DBD contains two zinc finger motifs that specifically recognize and bind, either as a monomer or homodimer, to the NGFI-B response element (NBRE, AAAGGTCA) within the promoter regions of target genes, thereby regulating their transcription (Crean and Murphy, 2021). Unlike classical nuclear receptors, the LBD of NR4A1 lacks a canonical ligand-binding hydrophobic pocket. However, it can still interact with ligands or small-molecule compounds through several hydrophobic amino acid residues, thereby modulating protein stability, subcellular localization, and transcriptional activity. To date, no endogenous ligand has been identified for NR4A1; accordingly, it is designated as an orphan nuclear receptor (Chen et al., 2020; Maxwell and Muscat, 2006). NR4A1 undergoes nucleocytoplasmic shuttling mediated by its nuclear localization signals (NLSs) and nuclear export signals (NESs), enabling rapid adaptation to changes in the extracellular milieu (Katagiri et al., 2000). This dynamic trafficking underpins its bidirectional regulatory functions observed across various disease models. Moreover, the combination of these shuttling motifs with a ligand-binding domain confers upon NR4A1 a dual capability that encompasses both nuclear transcriptional regulation and non-canonical signal transduction. Ultimately, the specific functional outcomes of NR4A1 are highly context-dependent, dictated by its subcellular localization and the prevailing pathological conditions.

### 2.2 Functional features of NR4A1

#### 2.2.1 Subcellular localization

The function of NR4A1 is primarily dependent on its subcellular localization. NR4A1, an orphan nuclear receptor, was initially considered to act primarily via nuclear transcriptional regulation. However, recent evidence from studies on hypoxic-ischemic brain injury reveals that in response to hypoxia and glucose deprivation, NR4A1 translocates from the nucleus to the cytoplasm and subsequently localizes to mitochondria in apoptotic cells. Upon reoxygenation, NR4A1 transcription is upregulated and the protein redistributes back to the nucleus (Moriyama et al., 2024). Therefore, NR4A1 undergoes dynamic translocation among the nucleus, cytoplasm, and mitochondria in response to diverse cellular stimuli and microenvironmental changes, thereby yielding a spectrum of functional effects that can be divergent or even opposing. In the nucleus, NR4A1 can act either by direct binding to NBRE sequences in target gene promoters through its DNA-binding domain, or by regulating gene expression via interactions with other transcription factors. However, upon stimulation, NR4A1 translocates to mitochondria, where it interacts with Bcl-2 on the outer mitochondrial membrane, triggering cytochrome c release and activating the mitochondrial (intrinsic) apoptotic cascade (Fu et al., 2025; Xinxing et al., (2012); Fechter et al., 2018). Cytoplasmic NR4A1 also contributes to the regulation of inflammation. In macrophages, NR4A1 functions as a lipopolysaccharide (LPS)-binding protein and cooperates with mitochondrial DNA to activate the non-canonical NLRP3 inflammasome, thereby regulating the inflammatory response (Zhu et al., 2023; Lith et al., 2024). In an inflammatory bowel disease model, NR4A1 not only represses NLRP3 and IL-1β transcription but also directly interacts with NLRP3. These proteins subsequently co-translocate to the Golgi apparatus, where their interaction blocks NLRP3 inflammasome activation and assembly, thereby conferring a protective effect against the disease (Deng et al., 2021).

#### 2.2.2 Post-translational modifications

Post-translational modifications often alter the transcriptional activity and stability of proteins. Upon stimulation by external cellular signals, NR4A1 is dynamically regulated by post-translational modifications such as phosphorylation and SUMOylation, which alter its subcellular distribution and DNA- or co-regulator-binding capacity, thereby modulating its transcriptional activity (Wang et al., 2023; McMorrow and Murphy, 2011). Phosphorylation is the addition of a phosphate group to a protein, typically on serine, threonine, or tyrosine residues. Studies have demonstrated that JNK upstream activators (e.g., TPA, anisomycin, and MEKK1) phosphorylate Nur77 and promote its nuclear exit, whereas kinase inhibitors block both this export and apoptosis (Han et al., 2006). Akt phosphorylates NR4A1 at Ser351, promoting its translocation from the nucleus to the cytoplasm, a phenomenon confirmed in 293 cells, NIH 3T3 cells, and H460 lung cancer cells (Kiriyama et al., 2025). Consistently, mutation of Ser354 to alanine, which blocks phosphorylation, inhibits NR4A1 nuclear export, whereas a phosphomimetic mutation to glutamic acid promotes nuclear export. Protein kinase C (PKC) can phosphorylate NR4A1 and induce its translocation from the nucleus to mitochondria. Notably, the S354A mutation impairs NR4A1 nuclear export and mitochondrial translocation in DO11.10 T cells, yet in 16610D9 CD4^+^ CD8^+^ cells it leads to constitutive localization to mitochondria and the cytoplasm. Thus, the nuclear-to-mitochondrial/cytoplasmic translocation of NR4A1 is both cell type- and stimulus-specific (Thompson et al., 2010). ERK2 phosphorylates NR4A1 at Ser237 and promotes its mitochondrial translocation.

Small ubiquitin-like modifier (SUMO) modification (SUMOylation) is a post-translational modification that affects protein stability and transcriptional activity. SUMOylation at lysine residues K102, K558, and K577 of NR4A1 not only induces its nuclear export and inhibits its transcriptional activity, but also promotes its degradation via the ubiquitin-proteasome pathway, thereby shortening its half-life and facilitating SP-induced autophagic cell death (Dodat et al., 2021; Zárraga-Granados et al., 2020). Therefore, PTMs critically regulate NR4A1 nucleocytoplasmic shuttling and the switch between its distinct functional modes.

## 3 The dual role of NR4A1 in renal fibrosis

### 3.1 Anti-fibrotic effects and mechanisms of NR4A1

#### 3.1.1 NR4A1 exerts anti-fibrotic effects via the TGF-β/Smad pathway

Transforming growth factor-β (TGF-β) is a major driver of renal fibrosis. In the injured kidney, TGF-β induces Smad2/3 phosphorylation and nuclear translocation, promoting the differentiation of quiescent fibroblasts into matrix-producing myofibroblasts. These myofibroblasts continuously secrete type I and III collagen, thereby driving epithelial-mesenchymal transition (EMT) and exacerbating the fibrotic response (Hoi et al., 2020; Zou et al., 2025). Current research has shown that NR4A1 modulates the TGF-β/Smad signaling pathway and exerts antifibrotic effects. In Nr4a1-deficient mice, the renal TGF-β/Smad signaling pathway is markedly activated, as evidenced by increased levels of p-Smad2/3 and type I collagen. Moreover, Nr4a1 enhances Smad7 protein stability in renal tubular epithelial cells, thereby suppressing sustained TGF-β/Smad signaling and alleviating tubulointerstitial fibrosis (Ma et al., 2022). In a mouse model of unilateral ureteral obstruction (UUO)-induced renal fibrosis, Nr4a1 overexpression significantly reduced α-SMA and type I collagen expression in HK-2 cells and inhibited TGF-β1-induced fibrosis. Further studies identified SPDEF as an upstream regulator of Nr4a1, capable of binding to four candidate sites on the Nr4a1 promoter to transcriptionally activate Nr4a1 and thereby inhibit renal fibrosis (Wang et al., 2024a). Given that both TGF-β/Smad signaling and NR4A1 function primarily through transcriptional regulation, the foregoing findings suggest that NR4A1 may exert its antifibrotic effects by modulating this pathway. However, direct evidence establishing a causal link between NR4A1 subcellular localization and TGF-β/Smad regulation in renal fibrosis models remains lacking to date.

#### 3.1.2 NR4A1 inhibits fibrosis by regulating the PI3K/AKT pathway

The phosphatidylinositol 3-kinase/protein kinase B (PI3K/AKT) signaling pathway is a key intracellular pathway involved in the EMT of renal tubular epithelial cells, and it is aberrantly activated in hypoxia- and hyperglycemia-induced renal fibrosis (Liu et al., 2025; Zhu et al., 2026; Zhang et al., 2021; Liu et al., 2017). TGF-β1 can activate the PI3K/AKT pathway as an upstream signal, synergistically promoting fibroblast proliferation and ECM deposition. In parallel, activated AKT phosphorylates downstream targets such as mTOR, SREBP1, and ICAM1, thereby enhancing inflammatory cell infiltration, inhibiting autophagy, and driving EMT in renal tubular epithelial cells together with cytoskeletal remodeling, ultimately leading to renal fibrosis (Wang et al., 2024b). Knockdown of Nr4a1 was shown to induce renal inflammation, promote apoptosis, and stimulate angiogenesis in UUO mice (Wang et al., 2024c). Subsequent studies confirmed that NR4A1 ameliorates UUO- or TGF-β1-induced renal fibrosis, an effect closely linked to suppression of the PI3K/AKT signaling pathway. NR4A1 may exert this effect by modulating PI3K or AKT expression, or by upregulating negative regulators such as PTEN, thereby reducing the activity of this signaling axis and suppressing EMT (Wang et al., 2024d). Notably, the interplay between NR4A1 and PI3K/AKT signaling is not unidirectional. Under certain pathological and stimulatory conditions, NR4A1 can also potentiate PI3K/AKT activity. Thus, the regulatory outcome likely depends on cell type, microenvironmental cues, and the functional state of NR4A1, and cannot be reduced to a single mechanism. Given its capacity for subcellular shuttling, NR4A1 may downregulate PI3K/AKT via its nuclear transcriptional activity; however, this localization-dependent mechanism warrants further investigation in renal fibrosis models.

#### 3.1.3 NR4A1 attenuates inflammation via suppression of NF-κB

Nuclear factor-κB (NF-κB) is a master transcription factor that governs renal inflammation. Its sustained activation drives macrophage infiltration, mesangial cell proliferation, and the release of pro-inflammatory cytokines such as TNF-α, IL-6, IL-1β, and MCP-1, thereby accelerating renal interstitial fibrosis and glomerulosclerosis. Mesangial proliferative glomerulonephritis (MsPGN) is an inflammatory nephropathy characterized by pathological mesangial cell (MC) proliferation and aberrant extracellular matrix (ECM) deposition. This condition can progress to glomerulosclerosis and interstitial fibrosis, ultimately leading to end-stage renal failure (Nihei et al., 2023). Nuclear NR4A1 functions as a transcriptional repressor by directly binding to NF-κB and inhibiting its transcriptional activity, or by competitively recruiting co-repressors to block NF-κB downstream gene expression, thereby attenuating the inflammatory cascade and mesangial proliferation. Bruceine A (BA), a bioactive compound isolated from the traditional Chinese medicinal plant Brucea javanica, binds to the D481/Q568 residues of NR4A1 and inhibits its ubiquitin-proteasome-dependent degradation. This stabilizes NR4A1 protein levels and enhances NR4A1-mediated transcriptional repression of NF-κB signaling, thereby attenuating the inflammatory response and mesangial proliferation and ultimately exerting a renoprotective effect (Hu et al., 2025). In a diabetic vascular injury model, a marked reduction in nuclear NR4A1 expression has been shown to similarly activate NF-κB signaling, thereby contributing to vascular damage in diabetes (Mao et al., 2026). Although the above studies support the anti-inflammatory and anti-fibrotic potential of NR4A1, much of the evidence originates from models of inflammatory nephropathy or other kidney injuries, rather than from classical renal fibrosis models. Therefore, the precise role of the NR4A1–NF-κB regulatory axis at distinct stages of renal fibrosis remains to be elucidated.

### 3.2 Pro-fibrotic effects and mechanisms of NR4A1 under pathological conditions

#### 3.2.1 NR4A1 promotes fibrosis via regulation of p38 MAPK

In renal fibrosis, TGF-β also activates non-canonical mitogen-activated protein kinase (MAPK) pathways, namely the c-Jun N-terminal kinase (JNK), extracellular signal-regulated kinase (ERK), and p38 subfamilies. In renal proximal tubular epithelial cells, TGF-β induces p38 MAPK activation, promoting the release of pro-inflammatory cytokines and ECM deposition, thereby accelerating renal interstitial fibrosis (Grynberg et al., 2017). Further studies have shown that ERK and p38 MAPK promote proteasomal degradation of the anti-fibrotic protein nuclear factor-erythroid 2 (NF-E2) and induce the expression of pro-fibrotic genes, including CTGF and FN, thereby driving fibrosis in renal proximal tubule cells (Li et al., 2021). The activation of p38 MAPK by Nr4a1 is closely associated with its cytoplasmic translocation. In the kidneys of UUO mice, NR4A1 expression is markedly upregulated and correlates positively with the severity of renal interstitial injury and the levels of fibrotic proteins. Treatment with the NR4A1 agonist cytosporone B (Csn-B) results in abnormal cytoplasmic accumulation of NR4A1 and induces p38 MAPK phosphorylation, thereby exacerbating UUO-induced renal interstitial fibrosis (Tao et al., 2023). This study suggests that, under pathological conditions, NR4A1 may regulate p38 MAPK signaling via changes in its subcellular distribution. However, given that the study predominantly assessed the correlation between altered NR4A1 localization and downstream signaling activation, whether cytoplasmic accumulation of NR4A1 directly drives p38 MAPK activation remains to be established.

#### 3.2.2 NR4A1 promotes fibrosis by modulating mitochondrial dynamics

Mitochondria are highly dynamic organelles that maintain normal morphology and function through a continuous balance of fission, fusion, and mitophagy (Fu et al., 2025). As an organ with high oxygen demand, the kidney is particularly rich in mitochondria. In tubular epithelial cells, ATP is generated via mitochondrial oxidative phosphorylation to sustain cellular energy production and metabolism. In chronic kidney diseases such as diabetic nephropathy, persistent hyperglycemia and associated oxidative stress disrupt mitochondrial homeostasis, leading to mitochondrial structural disorganization, swelling, functional defects, and impaired mitophagy. The electron transport chain is disrupted, ultimately promoting the accumulation of reactive oxygen species and impairing ATP production; persistent damage then triggers apoptosis and promotes renal fibrosis (Shao et al., 2026; Cleveland and Schnellmann, 2023; Li and Susztak, 2025). Studies have identified NR4A1 as a key regulator of hyperglycemia-mediated enhancement of mitochondrial fission and delayed mitophagy. In a diabetic nephropathy model, hyperglycemia-induced NR4A1 disrupts mitochondrial dynamics by activating the p53 signaling pathway and upregulating transcription of mitochondrial fission factor (Mff), thereby enhancing mitochondrial fission and exacerbating mitochondrial oxidative stress. Concurrently, mitochondrial translocation of NR4A1 promotes the opening of the mitochondrial permeability transition pore (mPTP), leading to the leakage of pro-apoptotic proteins such as cytochrome c into the cytoplasm and facilitating mitochondria-dependent apoptosis. Furthermore, mitochondria-localized NR4A1 inhibits Parkin-mediated mitophagy, preventing the timely removal of damaged mitochondria and resulting in their massive accumulation. This disruption of mitochondrial homeostasis exacerbates glomerular apoptosis and renal dysfunction. Thus, specific knockdown of Nr4a1, which blocks its mitochondrial translocation and downstream effects, reduces high-glucose-induced mitochondrial damage and apoptosis, thereby improving renal function and delaying the progression of renal fibrosis (Sheng et al., 2018). However, whether NR4A1 directly regulates the aforementioned processes via mitochondrial localization in kidney disease remains to be verified by subcellular fractionation and colocalization assays.

#### 3.2.3 NR4A1 mediates NLRP3 inflammasome activation to promote fibrosis

Renal tubular epithelial cells (TECs) are key players in the initiation and progression of renal fibrosis. Their injury, inflammatory responses, and phenotypic alterations contribute to the fibrotic microenvironment by releasing inflammatory mediators and fibrogenic factors, thereby accelerating fibrosis. However, renal fibrosis is a complex process involving multiple cell types, including fibroblasts, pericytes, endothelial cells, and immune cells, all of which also exert regulatory functions. Under hyperglycemic conditions, such as diabetic nephropathy, NR4A1 expression is markedly upregulated in HK-2 human renal tubular epithelial cells and may participate in fibrosis-related pathological processes by modulating NLRP3 inflammasome-mediated pyroptosis. Studies have shown that hyperglycemic stimulation upregulates NR4A1 expression in HK-2 cells, promoting NLRP3 inflammasome assembly. This in turn triggers caspase-1 activation, leading to the cleavage and release of IL-1β and IL-18, and simultaneously cleaves Gasdermin D (GSDMD) to initiate pyroptosis. Silencing NR4A1 suppresses NLRP3 activation and aberrantly activated PI3K/AKT signaling, alleviates high-glucose-induced pyroptosis in HK-2 cells, and downregulates the expression of fibrosis-related markers, including VIM, TGF-β1, CTGF, and COL1(46). Under pathological conditions such as hyperglycemia, NR4A1 may exhibit altered subcellular localization. In a macrophage model, cytoplasmic NR4A1 directly binds to NLRP3 and participates in inflammasome activation (Zhu et al., 2023). Therefore, in a high-glucose-induced injury model of renal tubular epithelial cells, NR4A1 may promote fibrosis-related responses by activating the NLRP3 inflammasome and pyroptosis. Together with evidence from other inflammatory models implicating cytoplasmic NR4A1 in NLRP3 regulation, these findings suggest that changes in NR4A1 subcellular localization may serve as a key determinant of its pro-inflammatory effects. However, this mechanism awaits direct validation through localization studies in renal fibrosis models.

### 3.3 Integration of mechanisms and resolution of contradictions in the dual regulation

The dual regulatory role of NR4A1 in renal fibrosis may be closely linked to its subcellular localization. Upon distinct stimuli, NR4A1 can shift its intracellular distribution, thereby modulating its transcriptional activity and interactions with signaling partners, which in turn leads to diverse functional outcomes. Under physiological or mild injury conditions, nuclear NR4A1 functions as a classical nuclear receptor, regulating the transcription of target genes involved in inflammation and fibrosis. In certain renal fibrosis models, NR4A1 suppresses pro-fibrotic gene transcription, stabilizes Smad7 to block TGF-β/Smad signaling, inhibits the PI3K/AKT axis, and antagonizes NF-κB activity, suggesting that its nuclear transcriptional function is associated with anti-fibrotic effects. Conversely, persistent pathological stimuli such as hyperglycemia and hypoxia may trigger post-translational modifications, including phosphorylation and SUMOylation mediated by Akt, PKC, and ERK2, that alter NR4A1 subcellular distribution, attenuating its nuclear transcriptional activity while promoting its cytoplasmic or mitochondrial functions. In other inflammatory models, cytoplasmic NR4A1 has been shown to interact with NLRP3 and participate in inflammasome activation, indicating that cytoplasmic localization confers functions distinct from its nuclear role. Moreover, mitochondrial NR4A1 may be involved in regulating mitochondrial dynamics, apoptosis, and autophagy; however, its specific contribution to renal fibrosis remains to be validated.

Of note, NR4A1 exerts bidirectional effects on PI3K/AKT signaling, providing a clue to its functional diversity. In UUO-induced renal fibrosis, NR4A1 suppresses PI3K/AKT hyperactivation to confer protection; however, under high-glucose metabolic stress, it may facilitate NLRP3 inflammasome activation and PI3K/AKT enhancement. The discrepant outcomes between UUO and DKD models imply that differential pathological states direct NR4A1 to distinct subcellular sites, which in turn determines its regulatory influence on PI3K/AKT. Hence, PI3K/AKT modulation is likely a downstream event of NR4A1 subcellular redistribution, not a direct target of NR4A1’s bidirectional actions.

Collectively, whether NR4A1 exerts anti-fibrotic or pro-fibrotic actions appears to depend on a complex interplay of cell type, environmental stimuli, and the signaling network equilibrium governed by its subcellular compartmentalization. Hence, strategies designed to fine-tune NR4A1 subcellular localization, thereby preserving its protective functions and curtailing detrimental signaling, may open new perspectives for targeted therapy in renal fibrosis.

The anti-fibrotic effects of NR4A1 in renal fibrosis models are summarized in Table 1.

**TABLE 1:** Anti-fibrotic effects of NR4A1 in renal fibrosis models.

References	Disease/Model	Effector cells	NR4A1 localization	Intervention	Pathway involved	Functional effect	Major limitations
(Ma et al., 2022)	Age-related renal tubulointerstitial fibrosis (mouse)	Renal tubular epithelial cells	Not directly detected (presumed nuclear)	NR4A1 knockout/overexpression	TGF-β/Smad (stabilizes Smad7, inhibits Smad2/3 phosphorylation)	Anti-fibrotic	NR4A1 subcellular localization not validated; age-related model may differ from acute fibrosis mechanisms
(Wang et al., 2024a)	UUO mouse/TGF-β1-stimulated HK-2 cells	HK-2 cells (human renal proximal tubular epithelial cells)	Not directly detected (SPDEF-mediated transcriptional activation, presumed nuclear)	NR4A1 overexpression/knockdown; SPDEF overexpression	NR4A1 transcriptional activation (upstream regulation by SPDEF)	Anti-fibrotic (reduces α-SMA and collagen I)	NR4A1 localization not directly confirmed by immunofluorescence or subcellular fractionation
(Wang et al., 2024d)	UUO mouse/TGF-β1-stimulated HK-2 cells	HK-2 cells	Not directly detected	NR4A1 knockdown; Csn-B (agonist)	PI3K/AKT (pathway activated upon knockdown)	Anti-fibrotic	PI3K/AKT inhibition mechanism inferred rather than directly demonstrated; NR4A1 nuclear localization not directly validated
(Hu et al., 2025)	MsPGN (mesangial proliferative glomerulonephritis)	Mesangial cells	Nuclear (BA stabilizes nuclear NR4A1 protein)	Bruceine A (binds NR4A1 D481/Q568, inhibits ubiquitin-proteasome degradation)	NF-κB (transcriptional repression)	Anti-inflammatory/anti-fibrotic	Evidence derived from inflammatory nephropathy, not classical renal fibrosis models
(Mao et al., 2026)	Diabetic vascular injury	Vascular endothelial cells	Nuclear (NF-κB activation upon reduced nuclear expression)	Targeting C1q	NF-κB	Anti-inflammatory	Evidence derived from vascular injury models, not direct renal fibrosis evidence

The pro-fibrotic effects of NR4A1 in renal fibrosis models are summarized in Table 2.

**TABLE 2:** Pro-fibrotic effects of NR4A1 in renal fibrosis models.

References	Disease/Model	Effector cells	NR4A1 localization	Intervention	Pathway involved	Functional effect	Major limitations
(Tao et al., 2023)	UUO mouse/TGF-β1 stimulation	Renal tubular epithelial cells	Cytoplasmic accumulation (post-Csn-B treatment)	Csn-B (agonist); NR4A1 siRNA knockdown; SB203580 (p38 inhibitor)	p38 MAPK phosphorylation	Pro-fibrotic	Correlation rather than direct causality between NR4A1 cytoplasmic accumulation and p38 MAPK activation
(Sheng et al., 2018)	STZ-induced diabetic nephropathy (mouse)	Glomerular cells/tubular cells	Mitochondrial (presumed, not directly validated)	NR4A1-KO mouse; NR4A1 knockdown	p53 → Mff (mitochondrial fission); inhibition of Parkin-mediated mitophagy	Pro-fibrotic (mitochondrial damage, apoptosis)	NR4A1 mitochondrial localization not directly confirmed by subcellular fractionation or immuno-electron microscopy; part of the mechanism (p53/Mff) is transcriptional and not fully dependent on mitochondrial localization
(Li et al., 2025)	STZ-induced DKD rat/high-glucose-stimulated HK-2 cells	HK-2 cells	Not specified (presumed cytoplasmic)	NR4A1 siRNA silencing	NLRP3 inflammasome activation; PI3K/AKT	Pro-fibrotic (via pyroptosis)	NR4A1 subcellular localization not directly validated in renal fibrosis models; evidence for cytoplasmic NR4A1 binding to NLRP3 derived from macrophage models
(Zhu et al., 2023) (indirect evidence)	Macrophage inflammatory stimulation model	Macrophages	Cytoplasmic	Inflammatory stimulation; NR4A1 intervention	NLRP3 inflammasome	Cytoplasmic NR4A1 acts as a NLRP3-binding protein to promote inflammasome activation	Non-renal fibrosis model; different cell type, not directly extrapolatable

The bidirectional regulation of the PI3K/AKT pathway by NR4A1 is summarized in Table 3.

**TABLE 3:** Bidirectional regulation of PI3K/AKT signaling by NR4A1.

References	Disease/Model	Effector cells	NR4A1 localization	Intervention	Pathway involved	Functional effect	Major limitations
(Wang et al., 2024d)	UUO	Renal tubular epithelial cells	Nuclear (inferred)	NR4A1 overexpression; Csn-B (agonist)	Transcriptional regulation (direct or indirect); PI3K/AKT inhibition	Anti-fibrotic	NR4A1 nuclear localization not directly validated
(Li et al., 2025)	High glucose/DKD	HK-2 cells	Cytoplasmic (presumed)	NR4A1 siRNA silencing	NLRP3 inflammasome → Caspase-1/IL-1β axis → indirect PI3K/AKT activation	Pro-fibrotic	NR4A1 subcellular localization not directly validated in renal fibrosis models

**FIGURE 1:**
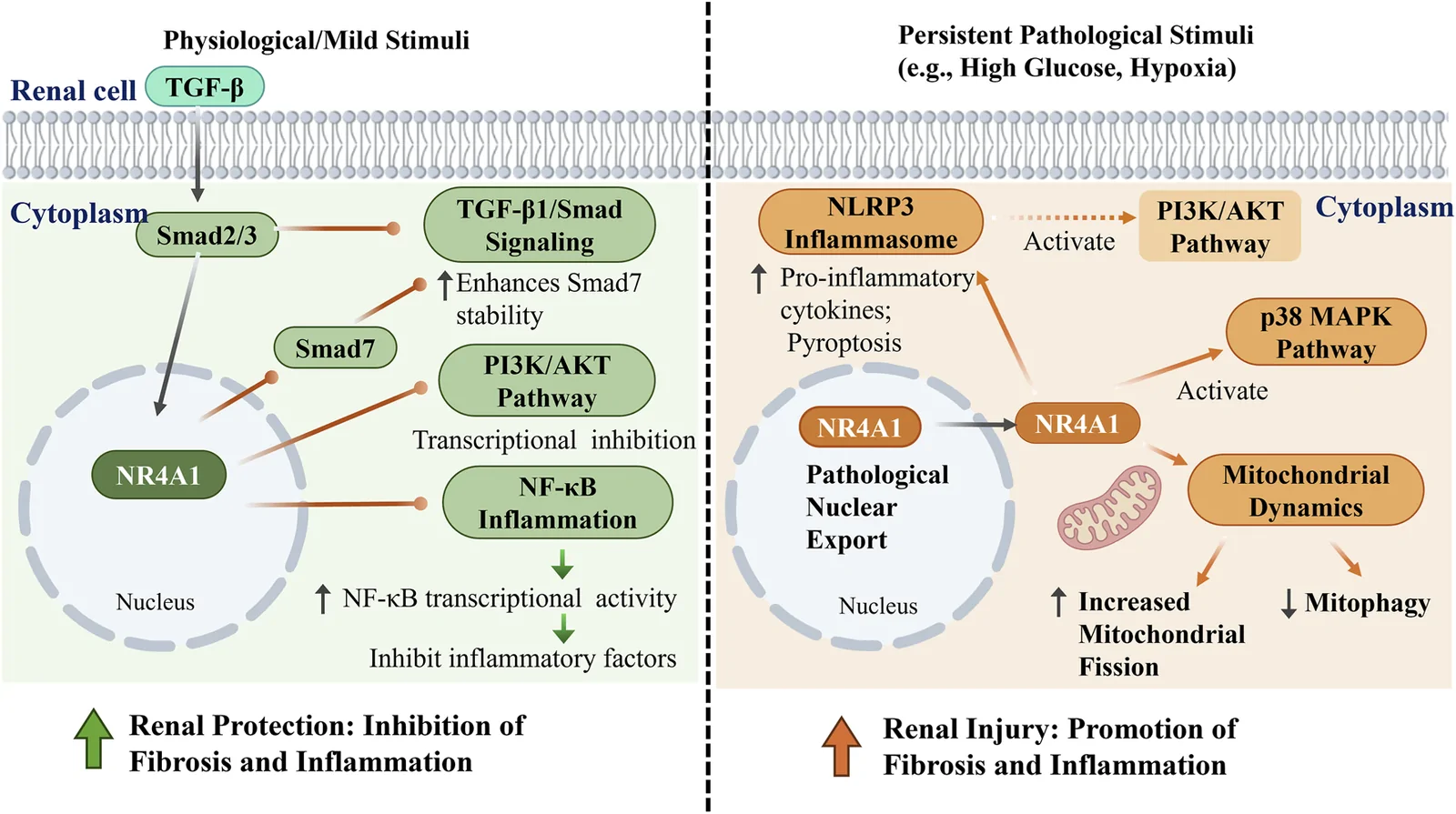
Dual regulatory mechanism of NR4A1 in renal fibrosis. Nuclear NR4A1 exerts anti-fibrotic effects by inhibiting TGF-β/ Smad, PI3K/AKT, and NF-κB signaling pathways. Upon pathological stimulation, NR4A1 translocates to the cytoplasm and mitochondria, where it promotes fibrosis by activating the NLRP3 inflammasome and p38 MAPK signaling and inducing mitochondrial damage.

This dual regulatory mechanism is summarized in Figure 1.

## 4 Strategies and challenges for NR4A1-targeted therapy of renal fibrosis

### 4.1 Agonists and antagonists of NR4A1

As an orphan nuclear receptor, NR4A1 is subject to modulation by small-molecule ligands or modulators. Cytosporone B (Csn-B), a commonly used NR4A1 agonist, has been explored in the context of inflammation, metabolic diseases, and tumors, and has been shown to induce fibrin expression and enhance NR4A1-mediated transcriptional regulation in renal fibrosis (Tao et al., 2023; Jiang et al., 2025). In a diabetic nephropathy model induced by unilateral nephrectomy combined with streptozotocin (STZ), the NR4A1 inhibitor DIM-C-pPhCO2Me upregulates fibrosis-related factors such as VIM and α-SMA in renal tissue and aggravates the fibrotic process (Wang et al., 2021).

However, given the bidirectional regulatory effects of NR4A1 across different renal fibrosis models, the use of its agonists or inhibitors must be tailored to the specific pathological context. When the protective functions of NR4A1 predominate, agonist administration may be beneficial. For instance, in UUO or TGF-β1-induced models, enhancing NR4A1 activity suppresses pro-fibrotic signaling, reduces inflammation, and decreases ECM deposition. Thus, activating the nuclear transcriptional function of NR4A1 may represent a potential strategy to potentiate its anti-fibrotic effects. Conversely, under persistent stress conditions such as hyperglycemia or hypoxia, NR4A1 may participate in NLRP3 inflammasome activation, mitochondrial dysfunction, and cellular damage responses. In these settings, the use of NR4A1 inhibitors may help suppress inflammatory responses and retard the progression of renal fibrosis. Therefore, the application of NR4A1 agonists or inhibitors should be evaluated on a case-by-case basis, taking into account the specific disease type and cellular context. Nevertheless, long-term use of non-selective agonists or inhibitors carries the risk of disrupting NR4A1-mediated normal inflammatory regulation, metabolic homeostasis, and immune function. Accordingly, agents that specifically target NR4A1 nuclear export may offer a more refined approach to harness its biological effects.

### 4.2 Targeting nuclear export and mitochondrial localization

Currently, no specific NR4A1 localization modulators are clinically available for renal fibrosis. However, several agents that regulate nuclear export or mitochondrial localization have been identified in non-renal fibrosis models, suggesting their potential therapeutic value in the kidney. In BGC-823 gastric cancer cells, treatment with the apoptosis inducer staurosporine (STS) promotes NR4A1 (TR3) translocation from the nucleus to the cytoplasm and subsequently to mitochondria, where it facilitates cytochrome c release and induces apoptosis (Zhan and Wu, 2004). In acute myeloid leukemia cells, fenretinide has been reported to trigger NR4A1 nuclear export and mitochondrial translocation, enhancing its interaction with Bcl-2, exposing the BH3 domain of Bcl-2, and exerting anti-apoptotic effects (Xiong et al., 2019). Notably, this anti-apoptotic action appears contrary to the pro-fibrotic role of mitochondrial NR4A1 in renal fibrosis, underscoring the need for validation in kidney fibrosis models. Furthermore, a study in cancer cells demonstrated that coumarin derivatives activate Jun N-terminal kinase (JNK) and p38 mitogen-activated protein kinase (MAPK), thereby inducing NR4A1–Bcl-2 interaction and promoting NR4A1 mitochondrial targeting (Zhou et al., 2014). These observations raise the possibility that agents modulating nuclear export, such as selective JNK or Akt inhibitors or activators, may hold therapeutic potential for renal fibrosis.

### 4.3 Prospects and translational challenges of nanoscale targeted delivery

Advances in nanomedicine have enabled the application of nanomaterials in both *in vitro* and *in vivo* studies for kidney disease treatment (Shang et al., 2024; Kianamiri et al., 2025). Active nanotargeting achieves kidney-specific delivery through ligand–receptor interactions by conjugating ligands, peptides, or antibodies to nanocarriers, thereby enabling precise drug distribution. Passive targeting, in contrast, exploits the unique physiological features of the kidney, such as its hemodynamics and glomerular filtration, to promote preferential renal accumulation through optimization of nanoparticle size, charge, shape, and material composition. Both strategies can achieve considerable therapeutic efficacy with minimal side effects (Cheng et al., 2024). In a unilateral ureteral obstruction (UUO) mouse model, nanoparticles loaded with the multi-kinase inhibitor sorafenib were specifically delivered to myofibroblasts, resulting in marked reductions in α-smooth muscle actin expression, myofibroblast infiltration, and collagen I deposition in the kidneys (Cheng et al., 2022). This highlights the therapeutic promise of nano-based delivery in renal fibrosis. In a cisplatin-induced acute kidney injury model, mitochondrial contents derived from nitric oxide-stimulated mesenchymal stem cell extracellular vesicles (pEVs) were precisely delivered to renal tubular cells, where they reduced mitochondrial reactive oxygen species and restored mitochondrial mass (Peng et al., 2025). Collectively, given that NR4A1 may exert distinct or even opposing effects in different cell types, conventional systemic administration carries the risk of nonspecific outcomes. The development of NR4A1-targeted mitochondrial delivery systems or nanoplatforms capable of precise drug delivery may therefore improve the safety and efficacy of NR4A1-based therapeutic strategies for renal fibrosis.

## 5 Conclusion

Renal fibrosis represents a common pathological pathway through which various chronic kidney diseases progress to end-stage renal disease, for which effective targeted therapies remain lacking. NR4A1 plays a dual role in renal fibrosis, exerting both anti-fibrotic and pro-fibrotic effects, with its functional orientation dictated by its subcellular localization and post-translational modifications. Therefore, NR4A1 is not a fixed pro-fibrotic or anti-fibrotic target, but rather a dynamic therapeutic node whose activity is governed by both its subcellular localization and the pathological microenvironment. This suggests that non-selective agonism or inhibition of NR4A1 is unlikely to precisely modulate its dual functions; instead, therapeutic strategies targeting its subcellular localization may hold greater promise. In recent years, carrier systems such as nanoparticle-based delivery vehicles have enabled precise subcellular drug delivery, demonstrating distinct advantages in antitumor immunity and anti-fibrotic therapy. However, the translation of these findings into clinical applications still faces substantial hurdles. These include an incomplete understanding of NR4A1 localization dynamics across different renal cell types and fibrotic stages, a lack of small-molecule modulators that specifically alter its subcellular distribution, and uncertainty regarding the optimal therapeutic window. To address these gaps, future investigations should integrate single-cell sequencing, spatial transcriptomics, and live-cell imaging to delineate the spatial and temporal relationship between NR4A1 localization shifts and its functional outcomes in distinct cell populations and disease phases. Such efforts will lay the groundwork for the development of cell-type-specific and stage-selective interventions, and ultimately facilitate their clinical translation.
